# Experimental and Numerical Response of Compressed Equal-Leg Steel Angle Members with Different Anchorage Lengths

**DOI:** 10.3390/ma19173801

**Published:** 2026-09-07

**Authors:** Ilona Szewczak, Małgorzata Snela, Jakub Paśnik

**Affiliations:** 1 Faculty of Civil Engineering, Lublin University of Technology, 40 Nadbystrzycka Str., 20-618 Lublin, Poland; m.snela@pollub.pl; 2 Department of Machine Design and Mechatronics, Faculty of Mechanical Engineering, Lublin University of Technology, 36 Nadbystrzycka Str., 20-618 Lublin, Poland; j.pasnik@pollub.pl

**Keywords:** steel angle members, compression members, end-fixture configuration, global buckling

## Abstract

Reliable tests of compressed steel members require appropriate representation of boundary conditions. This study examined the global response of compressed hot-rolled equal-leg steel angle members with three end-fixture configurations. Nine L50 × 50 × 4 specimens were tested with anchorage lengths of 50, 100, and 150 mm. Because increasing anchorage length reduced the unsupported member length, the observed differences reflect the combined effect of both parameters. Displacements and longitudinal strains were measured using Digital Image Correlation and strain gauges, respectively, and the tests were supplemented by finite element analyses. The configurations produced differences in maximum load and global displacement, although maximum load varied non-monotonically with anchorage length. At the investigated load levels, local longitudinal strains were similar, whereas global displacements differed. The K-5 specimens deformed in the direction opposite to the K-10 and K-15 specimens. Baseline numerical analyses reproduced the observed directions for K-10 and K-15, but not K-5. Reversing the sign of the initial imperfection reproduced the K-5 direction, supporting an interpretation involving two branches of the same global buckling mode; however, the cause of branch selection remained unresolved. The results show that end-fixture configuration should be considered when interpreting experimental and numerical assessments of compressed steel angle members.

## 1. Introduction

### 1.1. Influence of Actual Support Conditions on the Behaviour of Compressed Equal-Leg Angle Members

Hot-rolled equal-leg steel angles are widely used as bracing and compression members in buildings, trusses, lattice towers, and other lightweight steel structures. Their popularity results from simple fabrication, efficient transportation, and ease of connection. However, despite their apparently simple geometry, angle members exhibit complex stability behaviour. Equal-leg angles are open monosymmetric sections with relatively low torsional and warping stiffness, and their principal axes do not coincide with the geometric axes of the legs. Consequently, compressed angle members may exhibit flexural or flexural–torsional buckling. For sections with sufficiently slender legs, local plate buckling effects may also become relevant, and interactions between the different instability phenomena may occur [1,2,3,4,5].

The structural response becomes more complex when the load is introduced through only one leg. In such cases, the eccentricity between the load application line and the centroid of the cross-section generates additional bending moments, even when the member is nominally treated as axially compressed. Recent studies have therefore increasingly addressed angle members under combined compression and bending instead of assuming ideal concentric loading [1,2,6]. Differences between European, American, and other national design provisions further demonstrate that the prediction of the buckling resistance of angle members remains a non-trivial problem [1,2].

Experimental studies have shown that member slenderness, leg slenderness, material properties, load eccentricity, geometric imperfections, and end restraints all affect the observed response. Ban et al. [3] conducted buckling tests on 66 hot-rolled high-strength steel equal-leg angle columns covering a wide range of cross-sectional dimensions and member slenderness values. Spiliopoulos et al. [4] performed bending tests and 31 buckling tests on hot-rolled equal-leg angles subjected to concentric and eccentric compression. Huang et al. [5] investigated angle columns under concentric, mixed, and eccentric loading using different end-restraint devices. The results of these studies showed that the dominant instability response may change from flexural–torsional buckling to global flexural buckling depending on member slenderness, cross-sectional proportions, loading eccentricity, and boundary conditions.

### 1.2. Realistic Support Conditions in Experimental Investigations

In actual structures and laboratory tests, boundary conditions rarely correspond exactly to the idealised pinned or fixed restraints assumed in analytical models. The end connections of angle members may provide partial rotational restraint while simultaneously introducing load eccentricity, local deformation, contact effects, and additional bending moments. Therefore, the behaviour of the complete member–connection system should be considered when interpreting experimental results.

Reininghaus and Skottke [7] reported 40 tests on compressed equal-leg angle members with one-bolt end connections. Kettler et al. [8] subsequently assembled approximately 300 experimental results for compressed steel angles with bolted end connections and compared them with numerical calculations representing rigid and hinged supports of the gusset plates. Their study demonstrated that the influence of the gusset-plate support condition on member resistance could be more pronounced than the difference between one-bolt and two-bolt connections.

This conclusion was supported by a subsequent experimental programme comprising 27 compressed angle members with three end conditions: clamped, knife-edge, and fully hinged supports [9]. The study also included measurements of material properties and geometric imperfections, enabling detailed validation of nonlinear finite element models. The results showed that changes in the end-restraint system may produce substantial differences in the measured load-carrying capacity.

Using comparisons of European design procedures, worked examples, and experimental results available in the literature, Antonodimitraki et al. [10] quantified the influence of the number of bolts, gusset-plate thickness and support conditions, member slenderness, and steel grade on the predicted buckling resistance. Their results indicated that simplified assumptions concerning joint rigidity may not adequately represent the actual connection behaviour. A related design model was proposed by Kettler et al. [11], in which the restraint provided by practical bolted connections was represented by equivalent rotational springs. More recently, Vivek et al. [12] assessed updated IS 800 provisions for eccentrically loaded single-angle compression members, considering variations in member slenderness, cross-sectional slenderness, and end-connection and restraint configurations. Their comparison with published experimental results revealed marked differences in the predicted resistance, further highlighting the importance of appropriately representing the end conditions.

### 1.3. Numerical Assessment of Support Condition Effects

Numerical models provide an effective method for investigating the influence of boundary conditions, geometric imperfections, residual stresses, and loading eccentricity, provided that the models are validated against sufficiently documented experimental results. Bezas et al. [1] developed design rules for equal-leg angles subjected to compression and bending based on extensive experimental and numerical studies. Farhoud et al. [2] subsequently conducted geometrically and materially nonlinear finite element analyses to investigate the effects of the finite element formulation, the shape and amplitude of initial imperfections, and residual stresses. Their results showed that the adopted imperfection pattern and amplitude may affect the predicted member resistance.

Qu et al. [6] combined material tests, measurements of initial global and torsional geometric imperfections, 48 column tests, and finite element analyses to study high-strength equal-leg angles with eccentric single-leg connections. Their validated numerical models were subsequently used in parametric studies. Behzadi-Sofiani et al. [13] combined material testing, initial geometric imperfection measurements, member tests, and validated shell finite element analyses to investigate cylindrically pin-ended stainless steel equal-leg angle members under compression and major-axis bending. Their models were subsequently used in parametric studies covering hot-rolled and cold-formed members with a wide range of cross-sectional dimensions and member slenderness values. More recently, Nahushananda Chakravarthy et al. [14] tested 12 short hot-rolled steel equal-angle members with hinged end conditions and developed nonlinear finite element models for 72 angle sections. The numerical models were validated by comparing the ultimate loads and flexural–torsional failure modes with the corresponding experimental results.

Camotim et al. [15] compared the structural response of fixed-ended and simply supported angle columns, including members with cylindrically and spherically hinged end cross-sections. Their investigations highlighted the differences between various idealised support configurations and demonstrated that angle-specific stability models are required.

These studies show that numerical agreement with an elastic eigenvalue buckling mode alone is insufficient to validate a model. Reliable modelling requires an appropriate representation of material behaviour, initial geometric imperfections, residual stresses, load eccentricity, and the actual support configuration. Moreover, experimental results should be used to validate the numerical model, rather than numerical results being used to validate the experiment.

### 1.4. End-Fixture Anchorage Length as an Experimental Parameter

Previous investigations have considered idealised pinned and fixed end conditions [5,13,14,15], practical one-leg bolted connections [6,7,8,9,10,11,12], and purpose-designed end-restraint devices [5,9,15]. However, to the authors’ knowledge, the length over which the angle end is inserted into and restrained within a laboratory fixture has not previously been systematically compared or reported as an explicit descriptor of the fixture configuration. In the present arrangement, changing this length may affect load introduction and end restraint while simultaneously changing the unsupported member length. The observed response is therefore interpreted as the combined effect of these two changes rather than as the isolated effect of anchorage length.

In this study, the end-fixture anchorage length, *l_a_*, is defined as the length over which the angle end was inserted into and clamped within each purpose-designed end fixture. Owing to the fixture geometry, the anchorage length was equal to both the insertion depth of the angle member and the height of the clamping plates, and amounted to 50, 100, or 150 mm.

Increasing the end-fixture anchorage length may modify the translational, rotational, and torsional restraint provided by the fixture. However, because specimens of the same total length were tested, increasing the anchorage length simultaneously reduced the unsupported member length. Therefore, the separate effects of anchorage length and unsupported length cannot be distinguished on the basis of the present test series. Separating these contributions would require a two-factor experimental programme in which anchorage length and unsupported member length are varied independently. The effect of anchorage length could be investigated at a constant unsupported length by adjusting the total specimen length, whereas the effect of unsupported length could be examined using specimens of different total lengths while maintaining the same anchorage length and fixture geometry. Direct measurements of end rotations would additionally enable the restraint provided by the fixtures to be quantified.

### 1.5. Analytical Stability Assessment of Angle Members with Semi-Rigid End Restraints

The classical analytical description of the influence of end restraints on the elastic flexural buckling of a prismatic member is based on the effective buckling length. The corresponding elastic critical load may be expressed as:

(1) $$P_{\mathrm{cr}}=\frac{\pi^{2}\mathrm{EI}}{{\left(L_{\mathrm{eff}}\right)}^{2}}=\frac{\pi^{2}\mathrm{EI}}{{\left(\mathrm{kL}\right)}^{2}}$$

where:

*L*—unsupported member length;

*L_eff_*—effective buckling length;

*E*—Young’s modulus;

*I*—second moment of area about the considered buckling axis;

*k*—effective length factor.

In an idealized member with restrained end translations, the effective-length factor depends primarily on the rotational restraint provided at its ends. For a member with two pinned ends, (*k* = 1.0), whereas for a member with two fully fixed ends, (*k* = 0.5). Intermediate values may be associated with semi-rigid end restraints. Such restraints can be represented analytically by rotational springs with stiffness (*K_θ_*). Their influence may be expressed using a dimensionless stiffness parameter of the general form:

(2) $$\eta_{\theta }=\frac{\kappa_{\theta }L}{\mathrm{EI}}$$

Within this flexural idealisation and with the relevant end translations restrained, *K_θ_* approaching zero represents an ideal pin with respect to rotation about the considered buckling axis, whereas increasing *K_θ_* represents progressively greater rotational restraint. Experimental and numerical investigations have demonstrated that rotational restraint at the ends of compressed angle members can substantially influence their effective buckling length, deformation pattern, and load-carrying capacity [8,9,10,11].

However, the effective-length approach provides only a simplified description of the behaviour of angle members. Because equal-leg angles are open monosymmetric sections, their instability may involve flexural or flexural–torsional buckling, while local plate buckling effects may additionally become relevant for sections with sufficiently slender legs. Moreover, eccentric load introduction and interaction with the end fixtures may generate additional bending and torsional effects. Therefore, a value of (*k*) based solely on classical flexural buckling theory does not provide a complete description of either the actual end restraint or the resulting instability response.

The present study experimentally and numerically investigates the response of nine hot-rolled L50 × 50 × 4 equal-leg steel angle members of nominal steel grade S235 compressed in purpose-designed end fixtures. Three end-fixture anchorage lengths—50, 100, and 150 mm—were applied at both ends of specimens with the same total length of 1500 mm, resulting in nominal unsupported lengths of 1400, 1300, and 1200 mm, respectively. The response was evaluated in terms of ultimate load, midspan strain, three-dimensional displacement components, and the observed global deformation behaviour. The experimental results were compared with finite element analyses conducted in Abaqus.

The primary objective of the study is to compare the global response obtained for the three end-fixture configurations and to perform a comparative assessment of their consistency with the intended pinned-support idealisation. Because the anchorage length and unsupported length varied simultaneously, while the initial geometric imperfections and end rotations were not measured, the observed differences cannot be attributed solely to anchorage length. The results also do not permit a direct determination of the fixture rotational stiffness or proof of equivalence to an ideal pinned support. Any effective-length quantity inferred from the measured ultimate load is therefore treated only as an apparent comparative indicator. Within these limitations, the study addresses the identified research gap by treating end-fixture anchorage length as an explicit descriptor of the test configuration and by experimentally and numerically quantifying the differences in load-carrying capacity and global deformation response among the three configurations. The findings provide a documented basis for future experimental programmes in which anchorage length and unsupported member length are varied independently and end rotations and initial geometric imperfections are measured directly.

## 2. Laboratory Tests

**Figure 1:**
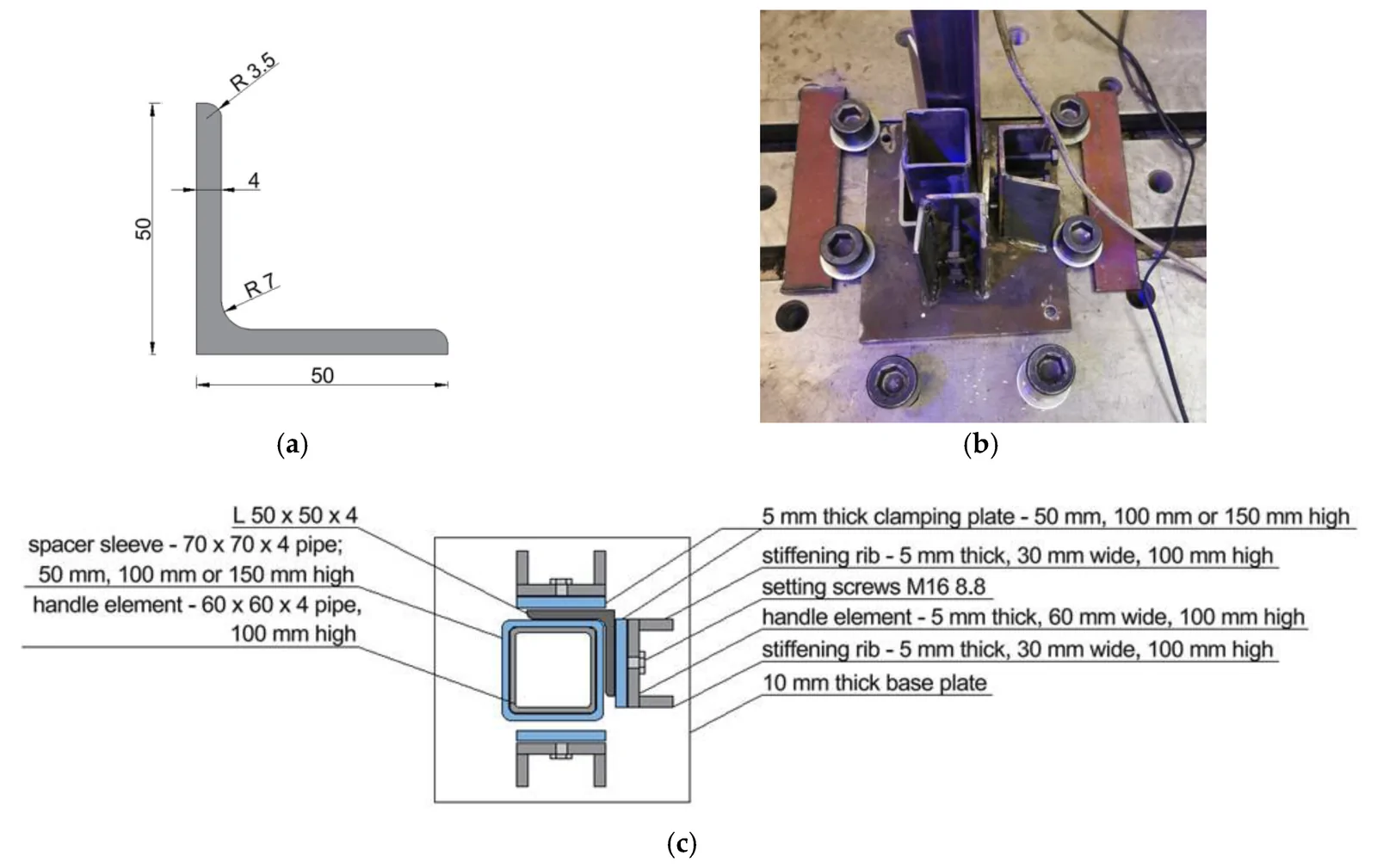
Geometry of the tested angle member and details of the end fixture: (**a**) nominal dimensions of the angle section; (**b**) close-up photograph of the end fixture; (**c**) top view of the end fixture with component labels.

The experimental investigation was conducted under conditions of axial compression in the laboratory of the Lublin University of Technology. Nine hot-rolled equal-leg steel angle members with a nominal cross-section of L50 × 50 × 4 and a total length of 1500 mm were tested. The specimens, fabricated from S235 steel, were divided into three groups of three, designated K-5, K-10, and K-15. Tensile coupon tests were not included in the experimental programme, and mill certificates for the tested angle members were not available. Consequently, the actual mechanical properties of the specimens could not be determined. The specimens were supplied as nominal-grade S235 steel. The nominal material properties adopted in the finite element analyses are reported in Section 4. The nominal cross-sectional dimensions are shown in Figure 1a, while the end fixture and its components are presented in Figure 1b,c.

Each end fixture comprised a 10 mm thick base plate, an inner 60 mm × 60 mm × 4 mm square hollow section (SHS) permanently welded to the base plate, and two mutually perpendicular reaction plates stiffened by triangular ribs. A replaceable 70 mm × 70 mm × 4 mm SHS spacer sleeve was fitted over the inner SHS. The end of the angle member was positioned against two adjacent outer faces of the spacer sleeve. Each angle leg was restrained by a 5 mm thick clamping plate pressed against the specimen using M16 grade 8.8 screws. The screws acted on the outer surfaces of the clamping plates and did not come into direct contact with the angle member.

The clamping plates and spacer sleeves had equal heights of 50, 100, or 150 mm, corresponding to the end-fixture anchorage length, *la*, over which the angle ends were inserted and restrained. The upper and lower fixtures were aligned with the loading axis of the testing machine, and the specimens were subjected to nominal axial compression. The specimen designations and corresponding fixture configurations are presented in Table 1.

**Figure 2:**
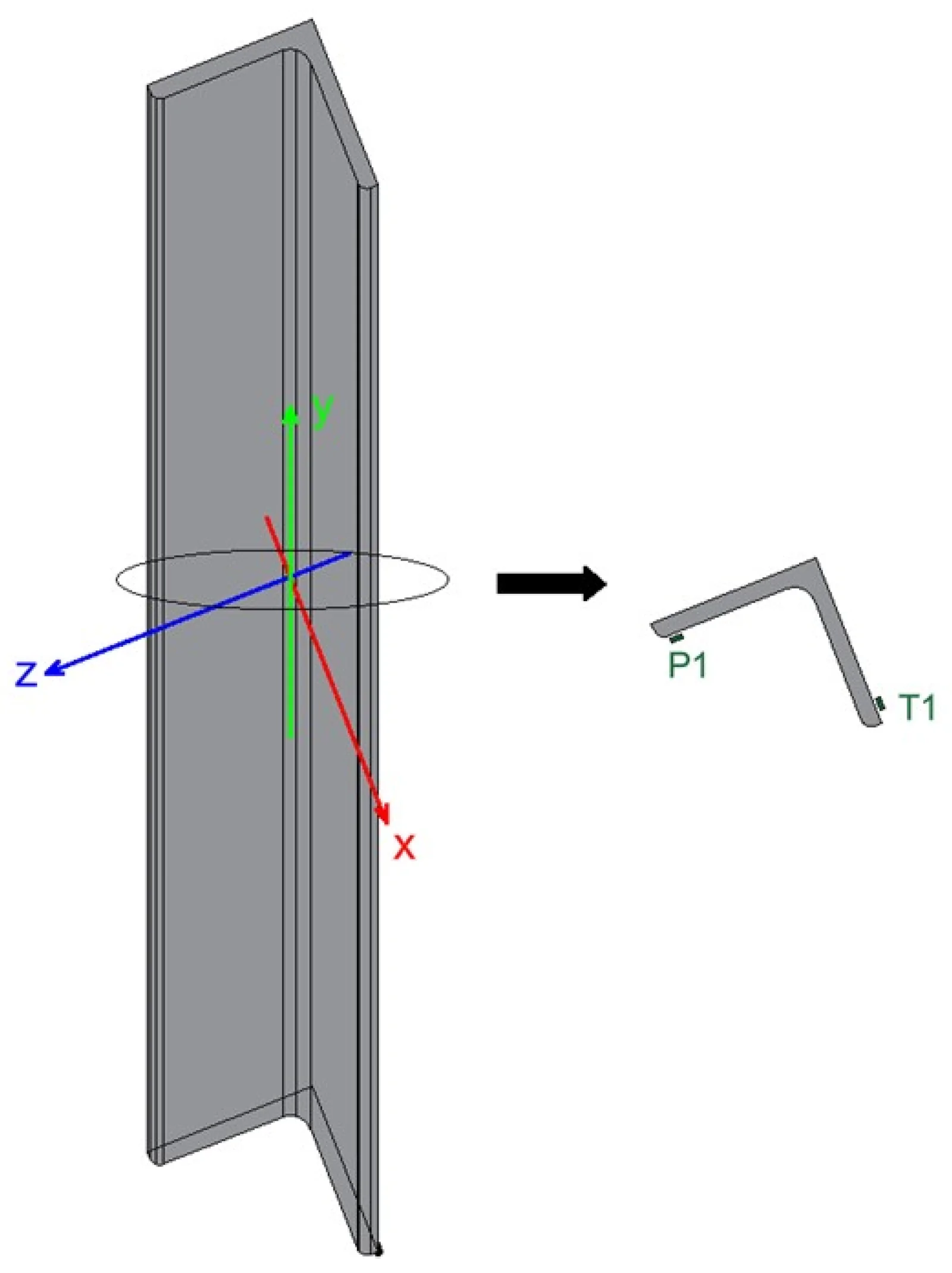
Location of the electrical resistance strain gauge (T1) and the displacement measurement point (P1) at midspan of the compressed angle members.

Prior to the commencement of the laboratory tests, all specimens were thoroughly cleaned. On each tested angle member, an electrical resistance strain gauge TENMEX TFs-10 (120 Ω ± 0.2%) was installed at midspan of the angle section, positioned 5 mm from the edge of the leg on the external surface of the profile in the y direction (see Figure 2). In addition, a measurement point was applied on the internal surface of the profile at midspan. These points were subsequently used by the Aramis optical measurement system to determine displacements. The location of the measurement point is presented in Figure 2.

**Figure 3:**
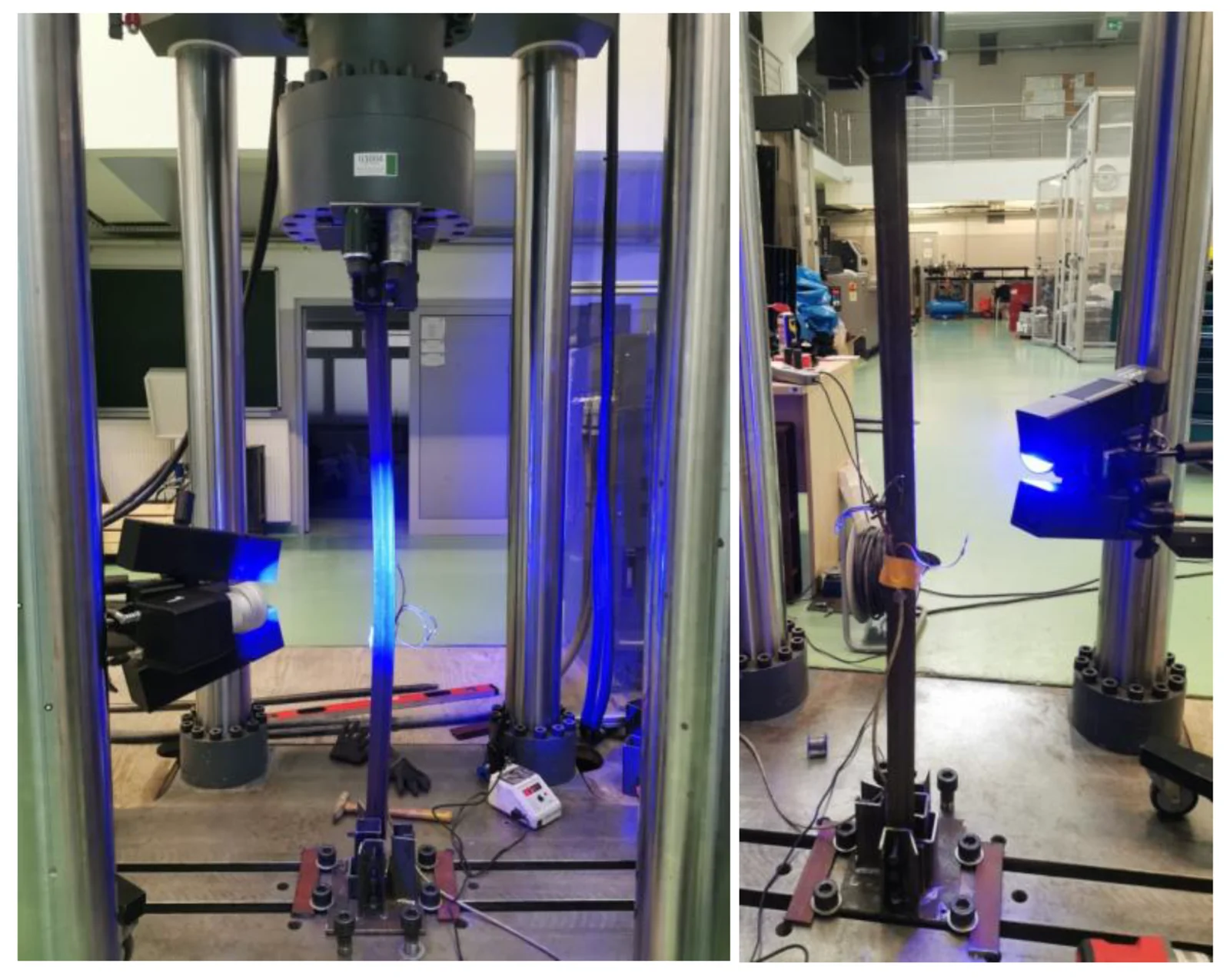
The test setup.

The specimens were mounted in the ZwickRoell testing machine (ZwickRoell GmbH & Co. KG, Ulm, Germany) using the corresponding upper and lower end-fixture configurations and subjected to nominal axial compression until failure. The tests were performed under displacement control at an actuator displacement rate of 3 mm/min. The load was recorded at intervals of 0.01 s (100 Hz). Longitudinal strains were measured using the electrical resistance strain gauge T1, while the three-dimensional displacements of the optical measurement target P1 were recorded using the ARAMIS system (GOM, GmbH, Braunschweig, Germany). Initial geometric imperfections, residual stresses, and specimen end rotations were not measured. The laboratory test setup is shown in Figure 3.

**Figure 4:**
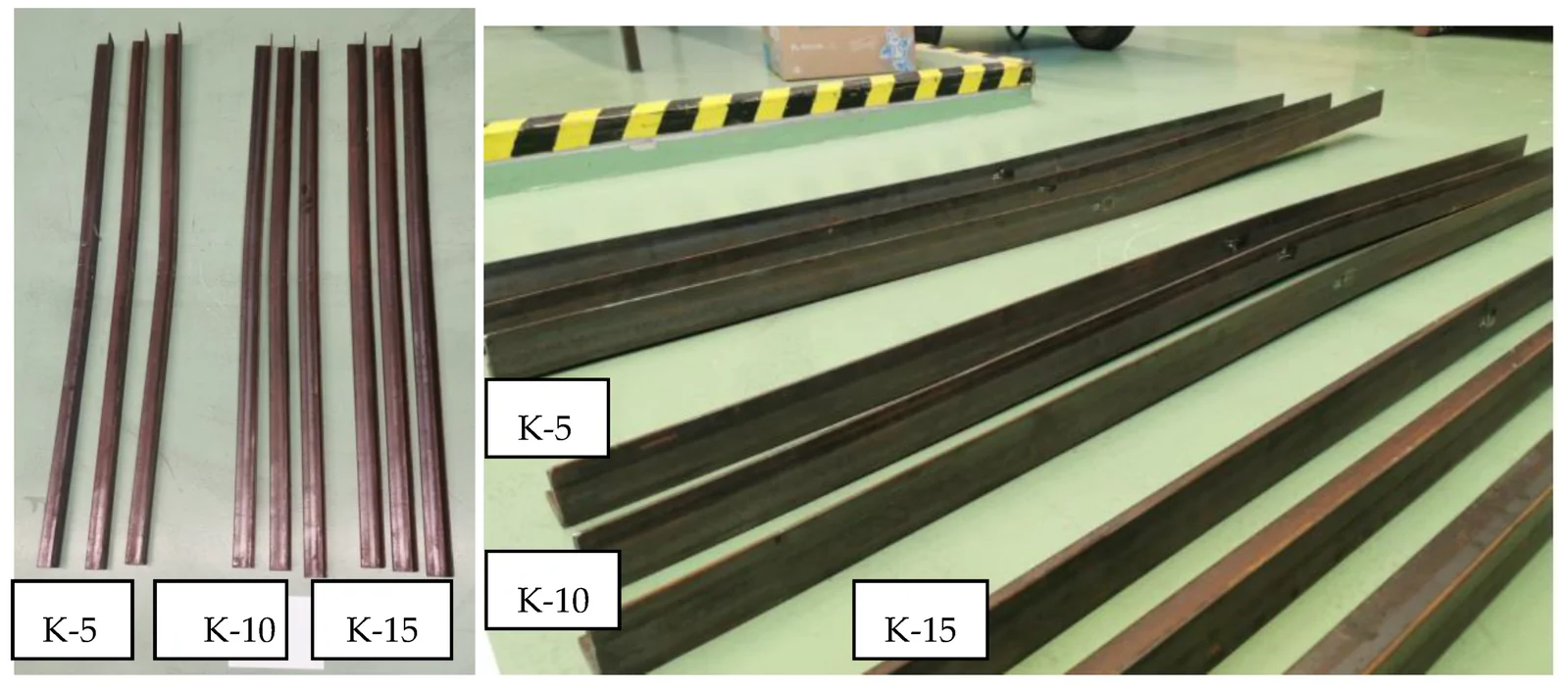
Post-test deformation shapes of the specimens.

The specimens exhibited a similar global buckling deformation pattern, although the deformation direction differed between the investigated end-fixture configurations. The K-5 specimens deformed in the direction opposite to that observed for the K-10 and K-15 specimens. The post-test deformation shapes are shown in Figure 4.

## 3. Experimental Results

**Figure 5:**
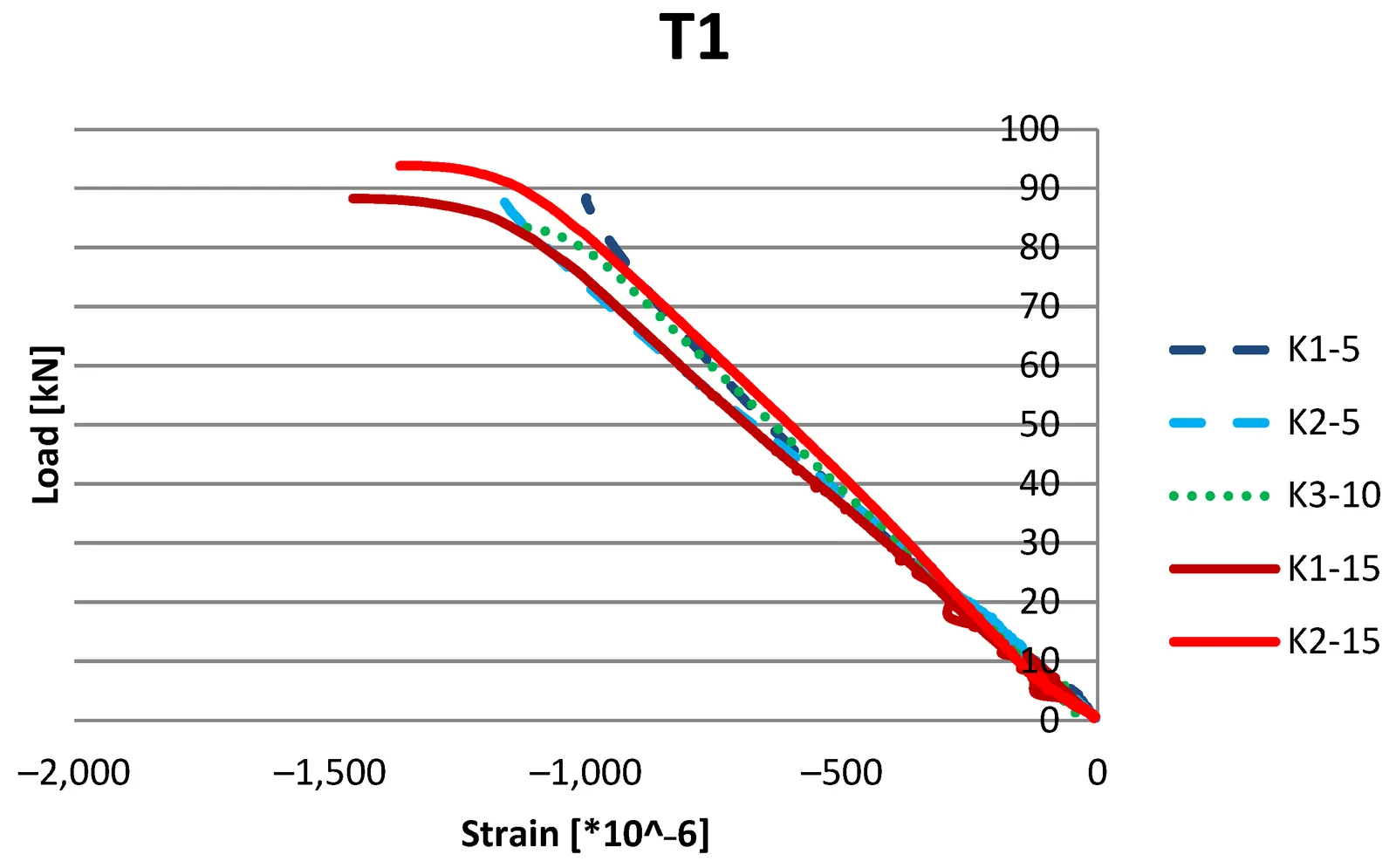
Load–strain relationship recorded at T1 for the tested specimens.

**Figure 6:**
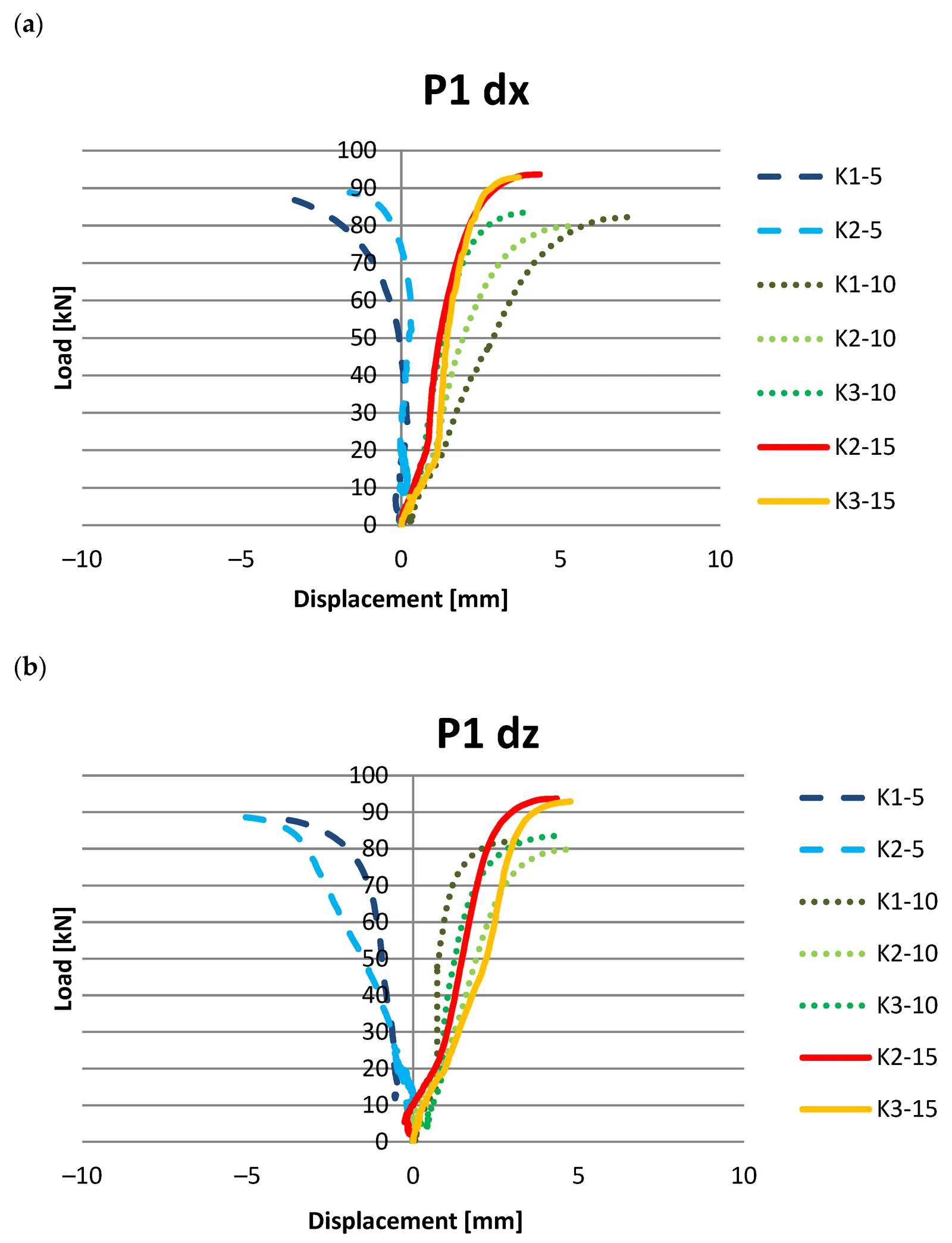
Load–displacement relationships recorded at P1 for the tested specimens: (**a**) displacement in the x-direction; (**b**) displacement in the z-direction.

During the laboratory tests, strain at T1 and the x- and z-components of displacement of the optical measurement target P1 were recorded for all specimens. The locations of T1 and P1 at midspan are shown in Figure 2. The load–strain relationships are presented in Figure 5, while the corresponding load–displacement relationships are shown in Figure 6.

To facilitate comparison of the test results, Table 2 presents the strain and displacement values recorded for the tested specimens at load levels of 60 and 80 kN. The final column provides the ultimate (failure) load for each angle member. The 60 kN load level was selected because the load–displacement responses of the specimens remained approximately linear up to this level. The 80 kN load level was selected as a common higher load level reached by all specimens included in Table 2, allowing their responses to be compared consistently. The symbol “x” denotes missing data resulting from strain-gauge malfunction or displacement-recording errors. Specimen K3-5 was excluded from Table 2 because, although the test was completed normally and the measurement signals were monitored in real time, the electronic data were not saved owing to a failure of the data-saving procedure. Global buckling was visually observed, but the load, strain, and displacement histories, including the ultimate load, could not be recovered.

To facilitate comparison between the investigated specimen groups, Table 3 presents the mean values calculated from the available valid test data for each group.

The mean values in Table 3 were calculated using only the available valid measurements. For group K-5, the strain, displacement, and ultimate-load values were based on two specimens because no retrievable measurement data were available for specimen K3-5. For group K-10, the displacement and ultimate-load values were based on three specimens, whereas the strain values represent only the single valid record obtained for specimen K3-10 and should not be interpreted as group averages. For group K-15, the strain and displacement values were based on two specimens, while the mean ultimate load was calculated from three tests. Consequently, the missing data reduce the statistical representativeness of the reported values, particularly for group K-5 and the K-10 strain measurements. The values in Table 3 should therefore be regarded as descriptive summaries of the available valid data rather than statistically representative group estimates.

## 4. The Numerical Model

The numerical simulations were performed using the finite element method (FEM) software Abaqus 2026 (Dassault Systèmes Simulia Corporation, Johnston, RI, USA). Numerical models of the angle members were developed to simulate their response under compressive loading. To obtain realistic simulation results that accounted for buckling, the numerical analyses were carried out in two stages [16]. In the first stage, the structure was subjected to a unit load, and the buckling modes were determined by the solver based on the model geometry, support conditions, and loading. This stage is commonly referred to as a linear eigenvalue buckling analysis. Once the buckling mode determined in the first stage had been compared with the actual behaviour of the angle member observed during the experimental tests, it was incorporated into the second, nonlinear stage of the numerical analysis. The input data for this stage included the cross-sectional dimensions, support conditions, loading, and the buckling mode determined in the first stage. During the nonlinear stage, the response of the angle member was simulated over the full loading range—from the beginning of load application until the loss of load-bearing capacity. The numerical problem was solved using the incremental–iterative Newton–Raphson method. The theoretical framework of this method is described extensively in [17,18].

More specifically, the first buckling mode, denoted as ϕ_1_ was used to define the initial geometric imperfection. In the baseline nonlinear analyses, the same sign convention, denoted as +ϕ_1_, was consistently adopted for all three end-fixture configurations. When interpreted in the experimental coordinate system, the corresponding eigenmodes exhibited the same deformation orientation. An additional sensitivity analysis was performed for group K-5 using the opposite sign, −ϕ_1_, while maintaining the same imperfection amplitude, to assess its influence on the predicted deformation direction.

The numerical models of the angle members were developed in Abaqus to accurately reproduce their geometry and simulate their response under compressive loading. Eight-node linear brick elements with reduced integration (C3D8R) were used. These general-purpose elements have a single integration point located at the centre of the element and three translational degrees of freedom at each node. Particular care was taken to ensure that at least three finite elements were used through the thickness of each angle leg to adequately represent its bending behaviour. Each of the three angle-member models comprised exactly 60,320 nodes and 48,504 linear hexahedral C3D8R elements. Particular attention was also paid to meshing the rounded corner of the angle section to reproduce the actual geometry of the specimen as accurately as possible. The dimensions of the angle section were adopted in accordance with Figure 1a.

The differences between the models lay in the way the ends of the profile were stiffened. Each model featured a stiffening component at the end of the angle member at a specified length—50 mm, 100 mm, and 150 mm on both sides. As described in the previous paragraph, the ends of the angle member were stiffened using steel plates pressed against the profiles. In the numerical model, these plates were simulated as non-deformable elements, since stresses and strains are not tested in them. The use of these elements reduces the time required to perform the simulation by eliminating the need for calculating stresses values at the nodes of the fastening elements. These factors improve the efficiency of working with the model, reduce the hardware load of the numerical calculations, and result in smaller output file sizes. The elements here are of R3D4 type—4-node, bilinear quadrilateral elements. The stiffening elements were connected to plates simulating the base and upper crossbeam of the testing machine. These plates provided support for the lower part of the angle member and applied a compressive load to the upper part. In the numerical model, these plates were integrated with the stiffening elements and were also meshed using non-deformable elements. Figure 1 shows that the stiffening elements were rigidly attached to the test specimens during experimental tests. The numerical model utilized a *Tie* constraint between the corresponding surfaces of the stiffening element and the test specimen in order to fully capture the nature of the connection between those components. In practice, the constraint mentioned above ensures that the two connected regions perform as a rigidly bonded structure throughout the numerical simulation. The stiffening of the angle member in the exemplary model with a length of 50 mm and the end plate is shown in the figure below.

**Figure 7:**
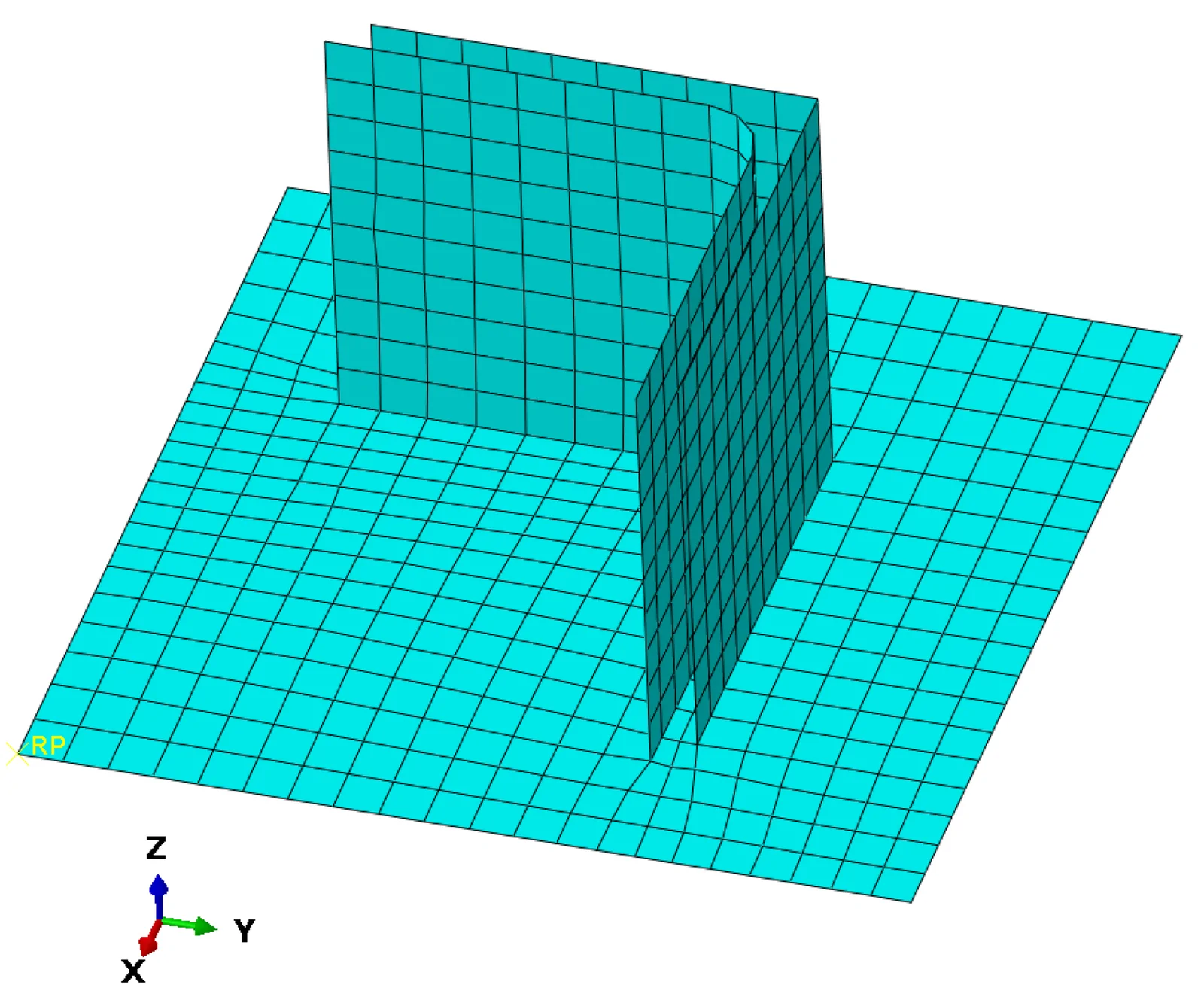
The end plate with a stiffening element—50 mm.

The results of numerical simulations depend to a large extent on the quality of the finite element mesh and the size of its elements. The mesh size used for the angle member was selected through an iterative procedure to achieve satisfactory agreement between the numerical and experimental results. A nominal element size of 4 mm was ultimately adopted, while maintaining at least three elements through the thickness of each angle leg. Further mesh refinement resulted in a substantial increase in computation time and computational resource requirements without providing a meaningful improvement in the accuracy of the numerical results. As demonstrated in [17], minor variations in mesh size do not significantly affect the obtained results (Figure 7).

**Figure 8:**
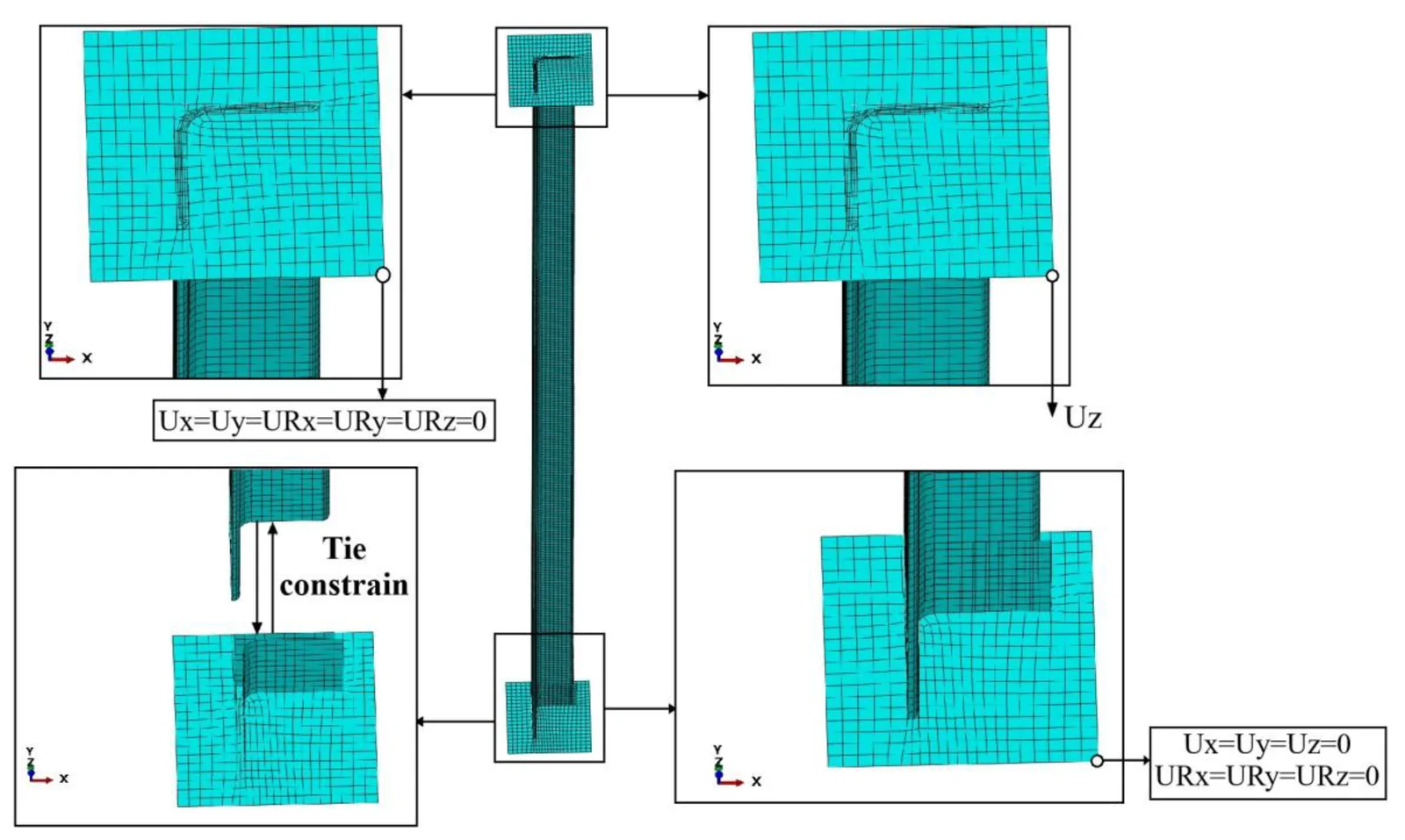
Boundary conditions and loading applied to the numerical model.

To perform the numerical analyses, the material behaviour was defined with consideration of the applied loading and the type of analysis. The nominal properties of S235 steel were adopted, with Young’s modulus *E* = 210 GPa, Poisson’s ratio ν = 0.30, yield strength *f_y_* = 235 MPa, and ultimate tensile strength *f*_u_ = 360 MPa. The material behaviour was represented using an elastic–plastic model. The angle member was connected to stiffening elements via the Tie constraint simulating a rigid connection between these elements. The boundary conditions included full fixation of the lower part of the support fixture and locking all degrees of freedom except for translation in the direction of the angle member axis of the top part. In this direction, displacement was forced to simulate the movement of the crossbar of a testing machine performing the compression process. The figure below shows the boundary conditions of the model (Figure 8).

## 5. Discussion of Experimental and Numerical Results

The experimental results demonstrate that the investigated end-fixture configurations were associated with differences in the global response of the compressed hot-rolled equal-leg angle members. However, their interpretation requires consideration of the simultaneous changes in two coupled parameters. Increasing the anchorage length may increase the rotational and torsional restraint provided by the fixture and modify the manner in which the load is introduced into the member. At the same time, it reduces the unsupported member length and hence the member slenderness, which would generally be expected to increase the global buckling resistance. Because these changes occurred simultaneously, the differences in maximum load and deformation response cannot be attributed exclusively to anchorage length. The non-monotonic variation in the mean ultimate loads further prevents the identification of a direct relationship between anchorage length and member resistance. The results should therefore be interpreted as a comparison of three complete end-fixture configurations characterised by coupled changes in anchorage length and unsupported member length, rather than as an isolated assessment of the influence of anchorage length.

Further insight into the support conditions is provided by the effective buckling length factors calculated using Equation (1). The values obtained for groups K-5 and K-15 were approximately 0.96 and 0.93, respectively. Both values are close to unity, indicating that the investigated end conditions remained predominantly pin-like. The slightly lower value obtained for group K-15 may suggest a modest increase in rotational restraint in the configuration with the greater anchorage length. However, the small difference between the calculated factors does not provide conclusive evidence of a systematic relationship between anchorage length and rotational restraint. Moreover, the results remain considerably closer to the ideal pin-ended condition (*k =* 1.0) than to the ideal fixed-ended condition (*k =* 0.5).

A notable observation emerges from considering the electrical resistance strain-gauge and Digital Image Correlation (DIC) measurements together. At the investigated load levels, the longitudinal strains recorded at measurement point T1 were similar among the analysed end-fixture configurations, whereas considerable differences were observed in the displacement components measured by DIC at point P1 (Table 2). This suggests that the effect of the combined change in anchorage length and unsupported member length was more apparent in the global displacement response than in the longitudinal strain recorded locally at T1. Consequently, changes in the end-fixture configuration may be reflected in the global deformation behaviour of the angle member without producing correspondingly large differences in the locally measured longitudinal strain. This interpretation should be treated with caution because complete strain-gauge data were not available for all specimens.

**Figure 9:**
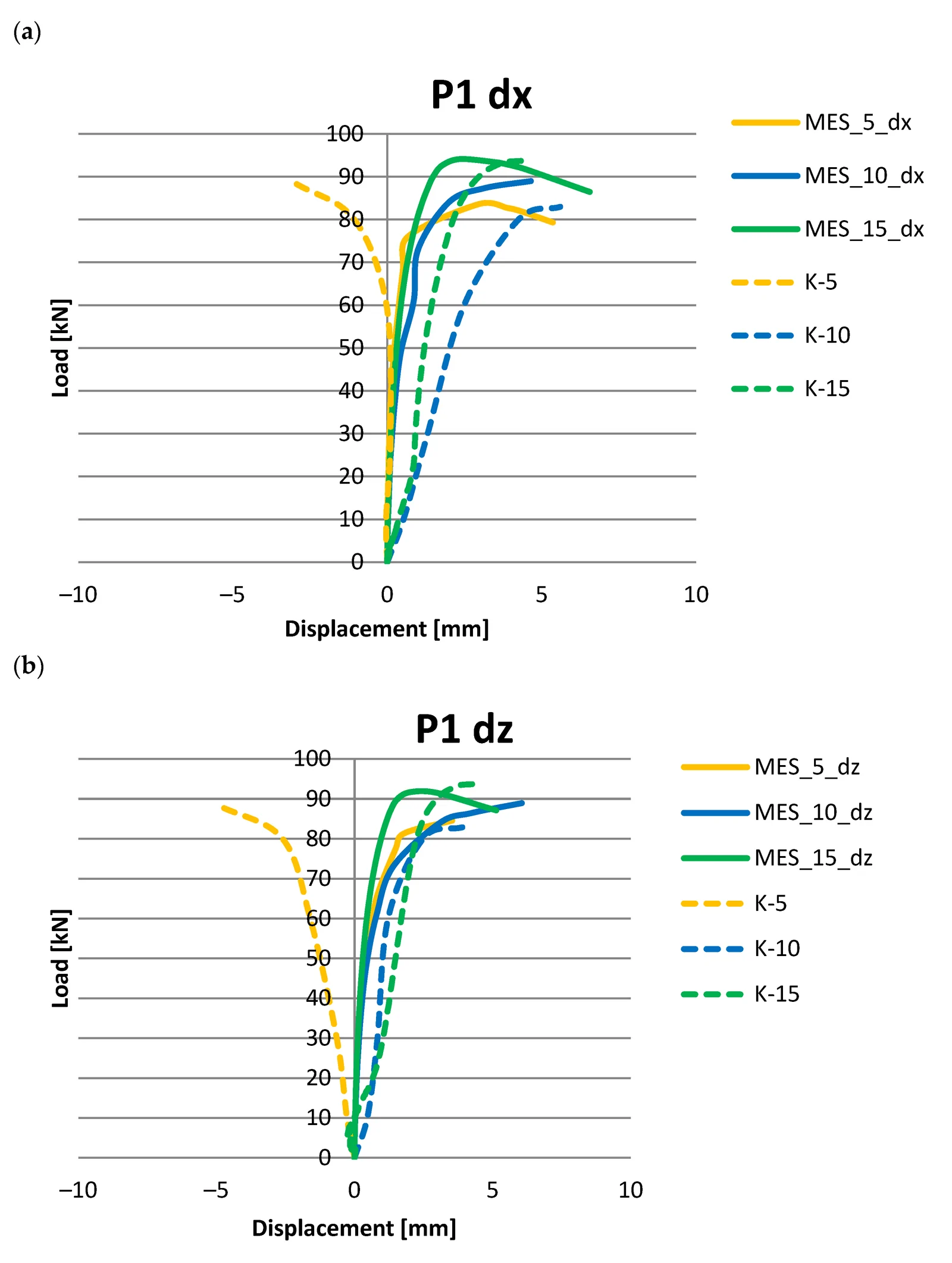
Comparison of the numerical and experimental load–displacement relationships for the investigated end-fixture configurations: (**a**) displacement in the x-direction; (**b**) displacement in the z-direction.

To assess the ability of the finite element models to reproduce the experimental response, numerical models representing all investigated end-fixture configurations were developed. A comparison between the averaged experimental results and the numerical predictions is presented in Figure 9.

In the baseline geometrically nonlinear analyses, the consistently adopted +ϕ_1_ imperfection reproduced the deformation directions observed experimentally for groups K-10 and K-15. For group K-5, however, the model predicted deformation in the same direction as for groups K-10 and K-15, which was opposite to that recorded during the laboratory tests. An additional analysis with the reversed imperfection, −ϕ_1_, resulted in deformation developing in the experimentally observed direction. Since +ϕ_1_ and −ϕ_1_ represent opposite signs of the same first global buckling mode, the numerical results are consistent with interpreting the observed deformation directions as two branches of the same global buckling mode rather than as different buckling mechanisms. Nevertheless, the model reproduced the experimentally observed direction for group K-5 only after the sign of the imperfection had been prescribed accordingly and therefore did not independently predict which deformation branch would be selected during the test. The reversed-imperfection analysis should therefore be regarded as a post-test sensitivity analysis rather than as an independent prediction of the buckling direction. Although it demonstrates that the model can reproduce both orientations of the same global buckling mode, the validation of the K-5 model remains conditional on prescribing the deformation branch observed experimentally.

Overall, the numerical curves reproduced the principal trends of the experimental load–displacement response for groups K-10 and K-15, although differences remained in the magnitudes of the individual displacement components. For group K-5, agreement in the deformation direction was obtained only after reversing the sign of the initial imperfection. A quantitative comparison of the experimental and numerical results is provided in Table 4.

As previously mentioned, the laboratory and numerical results exhibit the same overall trend. However, based on the data presented in Table 4, it is difficult to conclude that a satisfactory agreement between the obtained results has been achieved, as the differences in individual cases are in the range of approximately 50–60%. Nevertheless, no unambiguous conclusions can be drawn solely on this basis, as significant variations between individual specimens within a given series, as well as the limited number of specimens tested, should be taken into account. When comparing the results obtained for individual test specimens with those from the numerical analyses, a substantially higher level of agreement can be observed.

**Figure 10:**
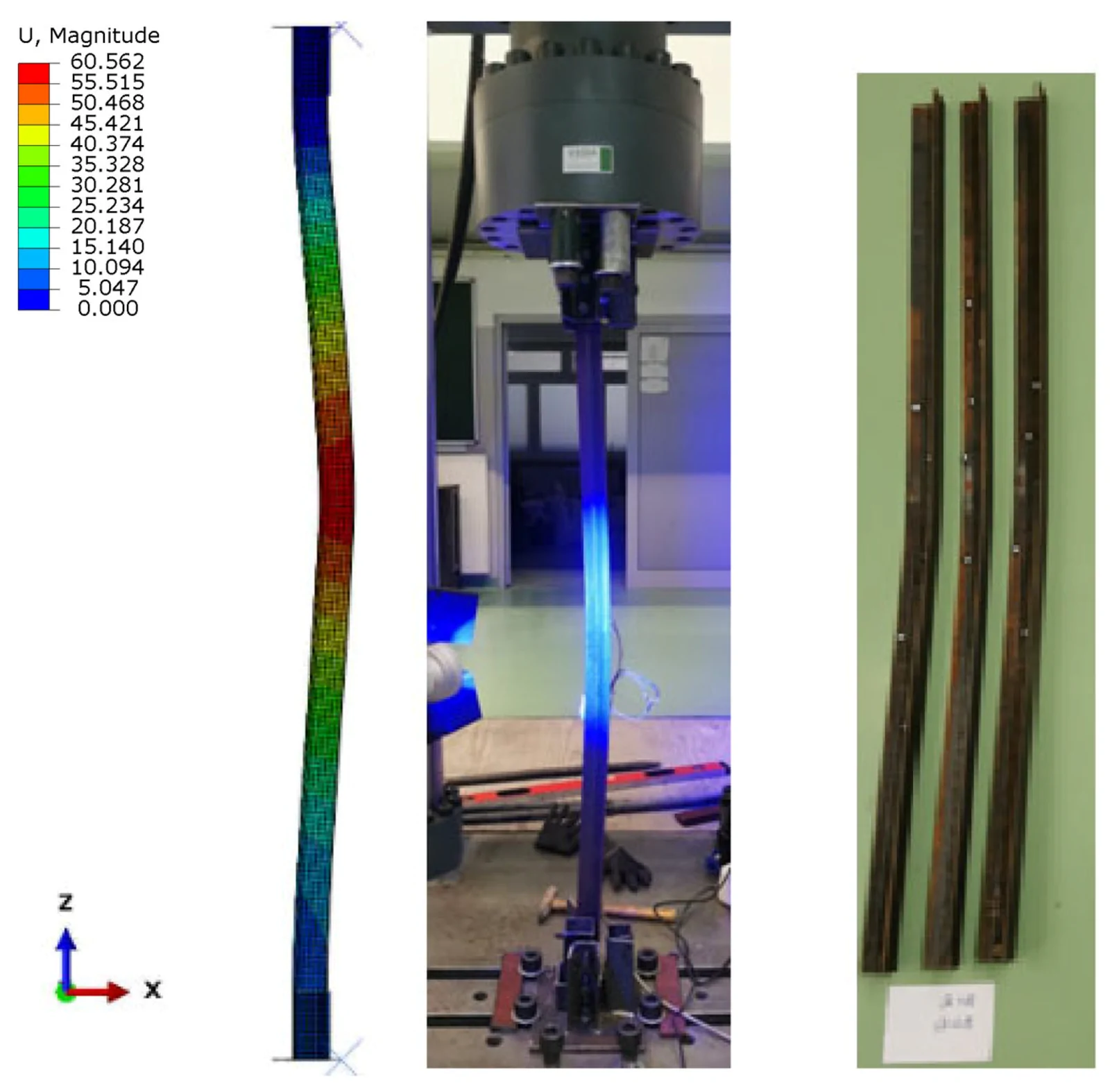
Comparison of the buckling deformation pattern predicted by the numerical analysis with that observed during testing and after unloading for a specimen from group K-10.

A further qualitative insight is provided by comparing the deformation pattern predicted by the numerical analysis with that observed during and after the laboratory tests, as shown in Figure 10.

Figure 10 shows good qualitative agreement between the numerical prediction and the experimental observations for a representative specimen from group K-10. Both the numerical model and the laboratory test exhibited a single half-wave along the member length, with a similar overall deformation distribution. The numerical model therefore reproduced the fundamental global buckling pattern observed experimentally for this group.

The selection of the deformation direction may have been governed by small asymmetries that were not represented in the numerical models, including the sign and amplitude of the initial geometric imperfections, load eccentricity, fixture misalignment, local contact and slip between the specimen and the fixture, or assembly-related effects. Because the reversal occurred consistently within the investigated specimen groups, it appears less likely to have resulted solely from random imperfections of individual specimens and may instead indicate a systematic difference associated with specimen preparation or testing conditions. However, as the initial imperfections and the detailed behavior of the fixture–specimen interface were not measured, the factor responsible for the observed direction cannot be identified conclusively.

Further contextual insight is provided by comparing the behaviour of group K-5 with the results reported in the authors’ previous studies [19,20]. As shown in Figure 4, the deformation direction observed for the K-5 specimens was similar to that reported for the compressed angle members investigated in those studies. In the previous experimental programme, the member ends were inserted into angle-shaped holes milled in 10 mm thick plates and rigidly restrained over an insertion length of 10 mm. However, this qualitative similarity does not demonstrate that the end conditions were equivalent, as the fixture geometry and restraint conditions differed between the experimental programmes. The maximum loads also differed. These differences may have resulted from the different end-fixture configurations, while the possible influence of material properties cannot be excluded because the specimens originated from different batches of steel. This comparison indicates that the deformation direction alone should not be interpreted as a direct indicator of the stiffness of the end fixture.

Overall, the investigation indicates that the tested end-fixture configurations influenced the global response of the compressed angle members. The combined changes in anchorage length and unsupported member length were accompanied by differences in the maximum loads, global displacements, and calculated effective buckling length factors. Nevertheless, the experimentally observed reversal of the deformation direction has not yet been fully explained. Further studies should include measurements of the initial geometric imperfections and end rotations, as well as numerical analyses considering both signs of the initial imperfection and a more detailed representation of fixture deformation, contact, friction, and slip.

## 6. Conclusions

The experimental investigation and numerical analyses presented in this study enabled an assessment of how the investigated end-fixture configurations affected the behaviour of compressed hot-rolled equal-leg steel angle members. Based on the obtained results, the following conclusions can be drawn:

- The investigated end-fixture configuration affected the global stability response of the compressed angle members. Differences were observed in the maximum load, global displacements, and post-buckling deformation response. However, the variation in maximum load was not monotonic with increasing anchorage length, as group K-10 exhibited a lower mean maximum load than groups K-5 and K-15.
- The specimens from group K-5 developed lateral deformation in the direction opposite to that observed for groups K-10 and K-15. This difference occurred despite the same nominal cross-section, material grade, specimen length, and loading procedure. Because the initial geometric imperfections and load eccentricities were not measured, the reversal cannot be attributed solely to the change in the end-fixture configuration.
- At the investigated load levels, the longitudinal strains recorded at measurement point T1 were similar among the investigated configurations, whereas the Digital Image Correlation measurements revealed differences in the global displacement response. This suggests that the effect of the fixture configuration was more apparent in the global displacement response than in the longitudinal strain recorded locally at T1. This interpretation should nevertheless be treated with caution because complete strain-gauge data were not available for all specimens.
- The finite element models reproduced the overall trend of the experimental load–displacement response and provided reasonably close predictions of the maximum loads for groups K-10 and K-15. The model also reproduced the single-half-wave global buckling pattern observed for the representative K-10 specimen. However, discrepancies remained in the displacement magnitudes, and the baseline analyses did not independently reproduce the experimentally observed deformation direction of group K-5.
- The linear buckling analysis yielded the same first global buckling mode for all investigated configurations. Reversing the sign of the initial imperfection corresponding to this mode caused the nonlinear numerical model to follow the opposite deformation branch. This supports the interpretation that the experimentally observed deformation directions may represent two branches associated with the same global buckling mode. However, changing the sign of the imperfection constitutes a prescribed numerical choice and does not explain why the K-5 specimens selected the opposite deformation direction during the experiments.
- The effective buckling length factors calculated with reference to the adopted member length were approximately 0.96 and 0.93 for groups K-5 and K-15, respectively. Their proximity to unity indicates that both end-fixture configurations produced a predominantly pin-like global response. The slightly lower value obtained for K-15 may suggest a modest increase in the effective end restraint. However, because the anchorage length and unsupported member length varied simultaneously, the difference cannot be attributed exclusively to increased rotational restraint.

The results demonstrate that the detailed end-fixture configuration can affect the global response of compressed equal-leg steel angle members and that an idealised representation of the end conditions may not capture all aspects of their behaviour. In particular, the opposite deformation direction observed for group K-5 was not independently predicted by the baseline numerical model but was reproduced in an additional analysis after reversing the sign of the initial imperfection corresponding to the first buckling mode. Further experimental studies should therefore include measurements of the initial geometric imperfections, load eccentricities, and end rotations, together with a more detailed numerical representation of the contact and restraint conditions within the fixtures. Such investigations would help determine whether the observed deformation direction was governed by the fixture configuration, specimen imperfections, loading eccentricity, contact effects, or a combination of these factors.

## Figures and Tables

**Table 1:** Method of marking the tested samples.

Specimen Designation	Specimen Group	End-Fixture Configuration
K1-5	K-5	Clamping-plate height: 50 mm Nominal unsupported length of the samples: 1400 mm
K2-5		
K3-5		
K1-10	K-10	Clamping-plate height: 100 mm Nominal unsupported length of the samples: 1300 mm
K2-10		
K3-10		
K1-15	K-15	Clamping-plate height: 150 mm Nominal unsupported length of the samples: 1200 mm
K2-15		
K3-15		

**Table 2:** Laboratory test data for individual specimens.

	Strain [*10^−6]		Displacement [mm]				Max. Load [kN]
			P1 dx		P1 dz		
Load	60 kN	80 kN	60 kN	80 kN	60 kN	80 kN	
K1-5	−751	−944	−0.34	−1.85	−1.07	−1.97	88.4
K2-5	−818	−1076	0.29	−0.28	−2.10	−3.23	89.1
K1-10	x	x	3.43	5.61	0.93	2.06	82.5
K2-10	x	x	2.41	5.35	2.26	4.78	80.1
K3-10	−754	−1010	1.54	2.71	1.50	2.86	83.8
K1-15	−815	−1080	x	x	x	x	88.3
K2-15	−724	−974	1.44	2.14	1.71	2.25	93.9
K3-15	x	x	1.61	2.17	2.46	2.96	93.1

**Table 3:** Mean values of the laboratory test results for each specimen group.

	Strain [*10^−6]		Displacement [mm]				Max. Load [kN]
			P1 dx		P1 dz		
Load	60 kN	80 kN	60 kN	80 kN	60 kN	80 kN	
K-5	−784.5	−1010	−0.03	−1.06	−1.59	−2.6	88.75
K-10	−754	−1010	2.46	4.56	1.56	3.24	82.13
K-15	−769.5	−1027	1.53	2.16	2.09	2.61	91.77

**Table 4:** Comparison of laboratory test results and numerical analyses.

	Displacement [mm]			
	P1 dx		P1 dz	
Load	60 kN	80 kN	60 kN	80 kN
K-5	−0.03	−1.06	−1.59	−2.6
FEM_5	0.39	1.82	0.64	1.69
K-10	2.46	4.56	1.56	3.24
FEM_10	0.82	1.5	0.78	2.47
K-15	1.53	2.16	2.09	2.61
FEM_15	0.44	0.96	0.45	0.99

## Data Availability

The original contributions presented in this study are included in the article. Further inquiries can be directed to the corresponding author.
